# Moving towards high density clinical signature studies with a human proteome catalogue developing multiplexing mass spectrometry assay panels

**DOI:** 10.1186/2043-9113-1-7

**Published:** 2011-02-08

**Authors:** Melinda Rezeli, Ákos Végvári, Thomas E Fehniger, Thomas Laurell, György Marko-Varga

**Affiliations:** 1 Div. Clinical Protein Science & Imaging, Biomedical Center, Dept. of Measurement Technology and Industrial Electrical Engineering, Lund University, BMC C13, SE-221 84 Lund, Sweden; 2 Institute of Clinical Medicine, Tallinn University of Technology, Akadeemia tee 15, 12618 Tallinn, Estonia; 3 First Department of Surgery, Tokyo Medical University, 6-7-1 Nishishinjiku Shinjiku-ku, Tokyo, 160-0023 Japan

## Abstract

A perspective overview is given describing the current development of multiplex mass spectrometry assay technology platforms utilized for high throughput clinical sample analysis. The development of targeted therapies with novel personalized medicine drugs will require new tools for monitoring efficacy and outcome that will rely on both the quantification of disease progression related biomarkers as well as the measurement of disease specific pathway/signaling proteins.

The bioinformatics developments play a key central role in the area of clinical proteomics where targeted peptide expressions in health and disease are investigated in small-, medium- and large-scaled clinical studies.

An outline is presented describing applications of the selected reaction monitoring (SRM) mass spectrometry assay principle. This assay form enables the simultaneous description of multiple protein biomarkers and is an area under a fast and progressive development throughout the community. The Human Proteome Organization, HUPO, recently launched the Human Proteome Project (HPP) that will map the organization of proteins on specific chromosomes, on a chromosome-by-chromosome basis utilizing the SRM technology platform. Specific examples of an SRM-multiplex quantitative assay platform dedicated to the cardiovascular disease area, screening Apo A1, Apo A4, Apo B, Apo CI, Apo CII, Apo CIII, Apo D, Apo E, Apo H, and CRP biomarkers used in daily diagnosis routines in clinical hospitals globally, are presented. We also provide data on prostate cancer studies that have identified a variety of PSA isoforms characterized by high-resolution separation interfaced to mass spectrometry.

## Introduction

Today's health care system is in a state of major restructuring and change. We envision a considerable shift in the paradigm of how and when we meet disease within the clinic due to both growing demand from an increasing number of patients as well as the ever escalating costs for providing resources to meet these needs. This is a global problem and actual shortcomings within our societies are realized on all continents and lifestyles.

For many common diseases, such as cancer, diabetes, neuro-degenerative and cardiovascular diseases there is an unmet need for diagnosing early indications of disease that could enable medical intervention and early treatment. At the same time as this is posed as one of the biggest challenges in modern health care, a novel opportunity is being created to build and generate a health care system that is driven by the medical research community with a patient-centric approach. This change in modern hospital infrastructure has already started, and is to a large extent a technology driven research commodity [1]. In this respect, we foresee that medical and biological mass spectrometry will continue to play a major role in the development new systems supporting health care, as well as within the development of new methods for monitoring efficacy and in developing new paradigms of targeted drug therapy. In order to be able to manage these goals, the understanding of disease pathophysiology and disease mechanisms, is a key component. The actual function of proteins, as well as expression alteration in disease in relation to healthy, is key in the understanding of disease evolvements, where bioinformatics plays a major role [2].

One approach taken to meet these needs for disease understanding is the establishment of clinical biobanks holding a variety of clinical samples from patients in diseased populations that have been clinically annotated and well characterized in terms of disease phenotype and outcome.

While some forms of common diseases can be managed effectively today, there is yet great unmet needs for effectively managing many forms of cancer, diabetes, obesity, infection and cardiovascular diseases. Together these represent a considerable number of cases requiring hospital based care and thus an ever increasing cost to society. For example chronic obstructive pulmonary disease (COPD) caused by smoking results in a loss of lung function and is now recognized as a major cause of debilitation and early death. As recently highlighted by the World Health Organization (WHO), COPD and lung disorders are exceptionally high in many regions of Asia [3]. Confounding medical care in diseases such as COPD is the lack of available drugs to slow down or inhibit disease progression. A further confounding factor is that COPD like other complex diseases involve many organ systems and often patients with COPD present with co-morbidities such as cancer and cardiovascular disease that also require other forms of medical intervention and modalities of treatment as well as methods for monitoring disease progression and efficacy. Protein expression databases and bioinformatics interoperations of protein functions, localization, as well as the link to clinical health care outcomes are currently a research area of great importance [4-7].

The recent developments and announcement from the Human Proteome Organization (HUPO), on the Human Proteome Project (HPP) is a major undertaking, in some ways similar to the Human Genome Project (HUGO). The major difference is that each of the approximate number of 20,300 proteins encoded by the human genome will mapped to specific locations on individual chromosomes. Protein annotations will be linked to the human genome and to specific diseases by applying both mass spectrometry assays and antibody based assays [8-10]. As such, this research project represents a major resource for the research community both now and for the future (announced at the 9^th ^Annual World Congress of the Human Proteome Organization, 19-23 September, 2010, Sydney, Australia; http://www.HUPO.org).

## Experimental

### Synthetic peptide standards

Light and heavy sequences of the target peptide with a purity higher than 97% were purchased from Thermo Fischer Scientific. The C-terminal Arginine or Lysine was labeled with ^13^C and ^15^N in the heavy forms.

### Sample preparation

K_2_EDTA-anticoagulating human blood plasma was used in all experiments. The seven highly abundant proteins were depleted in the plasma sample by using Plasma 7 Multiple Affinity Removal Spin Cartridge (Agilent Technologies). The first flow-through fraction was denatured, using 8 M urea in 50 mM ammonium bicarbonate buffer (pH 7.6). The proteins were reduced with 10 mM dithiolthreitol (1 h at 37°C) and alkylated using 40 mM iodoacetamide (30 min, kept dark at room temperature). Following buffer exchange with 50 mM ammonium bicarbonate buffer (pH 7.6) by using a 10 kDa cut-off spin filter (Millipore) the plasma samples were digested with sequencing grade trypsin (Promega) incubated overnight at 37°C. The plasma digest was spiked with a mixture of heavy isotope-labeled standards, and analyzed by nanoLC-ESI-MS/MS.

### LC-MS/MS analysis

LC-MS/MS analysis was performed on an Eksigent nanoLC-1D plus system coupled to an LTQ XL mass spectrometer (Thermo Fischer Scientific). Two μL of samples (0.02 μL plasma equivalent) were injected onto a 0.5 × 2 mm CapTrap C8 column (Michrom BioResources), and following on-line desalting and concentration the tryptic peptides were separated on a 75 μm × 150 mm fused silica column packed with ReproSil C18 beads (3 μm, 120 Å; from Dr. Maisch GmbH). Separations were performed at the flow rate of 250 nL/min in a 60-min linear gradient from 5 to 40% acetonitrile, containing 0.1% formic acid. One transition per protein was monitored. The parent ion was isolated with a mass window of 2.0 *m/z *units, fragmented (collision energy = 35%, activation time = 30 ms at *Q *= 0.25), and the resulting fragment ion was scanned in profile mode with a mass window of 2.0 *m/z *units. The maximum ion accumulation time was 100 ms, and the number of microscans was set to 1. The peak area responses were analyzed using Qual Browser, part of Xcalibur 2.0 software (Thermo Fischer Scientific).

## Biomarker Positioning and the Human Proteome Catalogue

**Figure 1:**
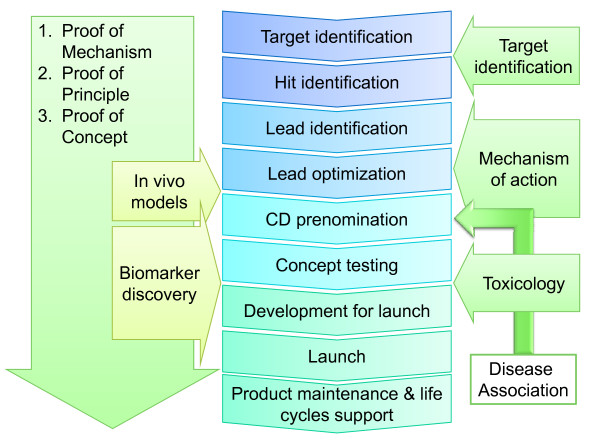
**Biomarkers within the Drug Development Process**.

A *biomarker *has been defined by the FDA working group, as: *"A characteristic that is objectively measured and evaluated as an indicator of normal biologic processes, pathogenic processes, or pharmacologic responses to a therapeutic intervention" *[11]. This definition of biomarker encompasses both molecular biomarkers as well as imaging modalities that can be used to describe the phenotype and stage of disease. As shown in Figure 1, protein biomarkers are importantly used throughout the entire drug development process, starting from target identification though to in vivo models of efficacy, through toxicology studies, and as safety markers. Recently, clinical studies with personalized drug related biomarkers have been presented [12], showing the effects of targeted receptor-ligand interactions, and their impact on cell signaling responses. As personalized drugs are being developed and are being positioned as a new generation of compounds with a clearly targeted mode of action, the use of biomarkers will be the natural link to monitor their use and effect. As a logical consequence and development after the delivery of the Human Genome Map in 2000 [13,14], the future of biomedical sciences focuses on understanding, the role of genome coded proteins. The follow up to these developments, experiences and strategic considerations was reported on recently [15,16].

Recently, the launch of the Human Proteome Project was made in Sydney at the 9^th ^HUPO World Congress, 23^rd ^September 2010. The Chromosome Consortium Project Outline was presented and approved by the General Council of the Human Proteome Organization (HUPO). The HPP initiative aims to develop an entire map of the Proteins encoded by the human genome that will be made publicly available. In the first part of the project, Protein sequences for each gene coded target protein will be determined and annotated. The initial ideas, strategies, and proclamation of sequencing and mapping the Human Proteome were presented recently by the HPP Working Group (http://hupo.org/research/hpp/) [10,17,18]. The HPP activities will surely play a central role in these developments, as a resourced facility where the basis of assay developments will be made available [19-21].

## Mass Spectrometry Based Protein Assay Technologies

Protein science as a research area, linked to the health care area, is adapting novel qualitative and quantitative measurements, based on new and improved technologies.

As such, the application of clinical proteomics has progressed considerably over the last few years, with the clear objective of helping determine early indications of disease and in monitoring disease progression and response to treatment. This focus also includes the understanding of disease links, virtually to any given target protein, or alteration in protein structure or function upon drug treatment. Patient safety and toxicity are also areas of expansion with a high priority in today's clinical and biomedical development. As outlined in the work stream presented in Figure 1, these activities have a solid biomarker link. The usefulness and interest in developing methodologies and assays intended for patient diagnosis and diagnostic application of protein analysis is a priority that is increasing both in demand but also a response too that demand. Advancing protein analysis for clinical use is aimed towards prognostic diagnostics, and biomarkers, where proteins have been used as markers of disease in clinical studies for more than a decade [22].

Advancing protein analysis for clinical use is aimed towards prognostic diagnostics, and biomarkers, where proteins have been used as markers of disease for more than a century. A major reason for the fast development within this field is greatly owed to the improved technology that has been made within the mass spectrometry field. This has happened in conjunction with new enabling tools and methods for quantitative proteome analysis. Liquid chromatographic separation interfaced with mass spectrometry has become the workhorse technology platform, which currently is the most dominant protein-sequencing engine within clinical proteomics today. The rapid progress within the field can be identified through the large number of clinical studies undertaken, as well as the fact that the data output, both in terms of depth and width is increasing rapidly. Today, medium abundant, as well as parts of the low abundant protein expression concentration regions can be addressed in clinical studies, using minute amount of clinical samples, such as blood fractions and tissue extracts [23,24].

But, there are unmet needs in terms of instrumentation and diagnostic validation capability that also are in demand for improving health care area. These limitations already extend from early indicators of disease, through disease severity, progressive disease development, and on to therapeutic efficacy. It is also interesting to note that an important source of these demands is the switch to personalized medicine approaches coupled with selective drug therapy both with small molecules and as well, by protein-based biopharmaceuticals [25].

### Multiplex Biomarker Assay Platforms - SRM

The assay principle is generic in a sense that it allows for any target protein sequence to be selected for assay development and measurement. SRM utilizes isotope labeled protein sequences used as internal standards, and the assay principle is operated without the use of antibodies - SRM is an immune-reagent-less technology that allows multiple biomarkers to be measured in a single cycle. The assay format can be built for many hundred of protein biomarkers, but practically with analytical performance and rigidity, the multiplex number is aimed at about 100 individual biomarkers. The high throughput capacity of such SRM-platforms is aimed at 10,000 quantitative assay points/day.

Selected Reaction Monitoring (SRM), also referred to as Multiple Reaction Monitoring (MRM), is a new mass spectrometry assay platform that quantifies multiple protein biomarkers in clinical samples in an assay cycle [26-28]. SRM is the current IUPAC definition standard for: "data acquired from specific product ions corresponding to *m/z *selected precursor ions recorded via two or more stages of mass spectrometry", whereas MRM is a company trade mark and not recommended by IUPAC.

Upon the development of an SRM assay, the selection of specific proteotypic peptides, representing the target biomarker proteins is crucial. Choosing the targeted peptides, can be based on both empirical data from shotgun experiments as well as utilizing the computational tools, like on-line data repositories (Peptide Atlas, GMP Proteomics database, PRIDE) that are available predicting the most likely observable peptide sequences.

SRM allows absolute quantification of a large set of proteins in complex biological samples with high accuracy, by the addition of isotopically labeled peptides or proteins, as internal standards. The quantification is based on the relative intensity of the analyte signal, compared to the signal of known levels of internal standards. These assay formats are usually applied, when any given concentration of a resulting outcome is assigned to a disease/health status. SRM assays are also developed for relative quantitation analysis, where internal isotope standards are not needed. This label-free assay format is typically applied to studies where the expression comparison in-between two sample types are to be compared. In these measurements, the absolute concentration is not of vital importance for the biological/clinical relevance. An example to this would be the relative comparison of EGF-Receptor expression difference in disease state, in relation to healthy controls.

Normalization is an important part of utilizing SRM assays and platforms for quantitative clinical analysis. In this respect, quality control (QC) samples are introduced in the cycle of analysis, and runs. We typically use one QC sample in an analysis cycle of 5 samples, and end the cycle by the analysis of an additional QC. A given statistical standard deviation window will be tolerated, *e.g.*, 10%, in-between the two QC samples. If the variation is outside the given criteria, the samples need to be normalized. The normalization is typically performed both in terms of retention time index, as well as signal intensity.

In addition, isotopically labeled internal standard peptides are not only useful in quantification but also in validation of the transitions. Regarding the issues, which relate to false positives in clinical analysis by the SRM platform, we are able to apply multiple-fragment monitoring, whereby the target peptide of the given biomarker is ensured.

In addition, heavy isotope labeled peptide co-elute with the endogenous target peptide, which also aids in avoided false positive annotations.

### SRM Applications

The cardiovascular disease area is in many sectors one of the most resource demanding challenge for the health care area, both in monitoring and treating disease. It is also the major disease area that requires assay-demanding activity, for clinical chemistry units at all major hospitals. We have developed a multiplex SRM assay where we have been able to align ten common markers that are typically quantified in an everyday clinical operation, as indicated in Table 1. The table also provides details on the specific amino acid and its position, where the isotopic labeling has been introduced. Typical clinical concentration ranges has been given in blood, where most patients fall within. Thus, it should be emphasized that these levels might be altered in diseases, by up-, or down-regulations that will impact on the data presented in Table 1.

**Table 1:** Protein markers typically monitored in clinical measurements.

Protein	Concentration in plasma	Target peptide
**Apo A1**	1-2 mg/ml	ATEHLSTLSEK*
**Apo A4**	0.13-0.25 mg/ml	SLAPYAQDTQEK*
**Apo B**	0.5-1.5 mg/ml	TEVIPPLIENR*
**Apo CI**	40-80 μg/ml	EWFSETFQK*
**Apo CII**	20-60 μg/ml	TYLPAVDEK*
**Apo CIII**	60-180 μg/ml	GWVTDGFSSLK*
**Apo D**	50-230 μg/ml	NILTSNNIDVK*
**Apo E**	20-75 μg/ml	LGPLVEQGR*
**Apo H**	71-380 μg/ml	ATVVYQGER*
**CRP**	1-5 μg/ml	ESDTSYVSLK*

Today, the multiplex SRM sensitivity limitation of a given protein is in the low ng/mL [27,29]. In the case of lower concentration regions, *e.g.*, in human blood samples, we need to introduce an enrichment step that will increase the signal intensity. Typically, large sample volumes can be applied, followed by extraction or immuno-affinity isolation, using an antibody probe [30]. Sensitivities down to pg/mL levels have been reported on applying these sample preparation technologies.

**Figure 2:**
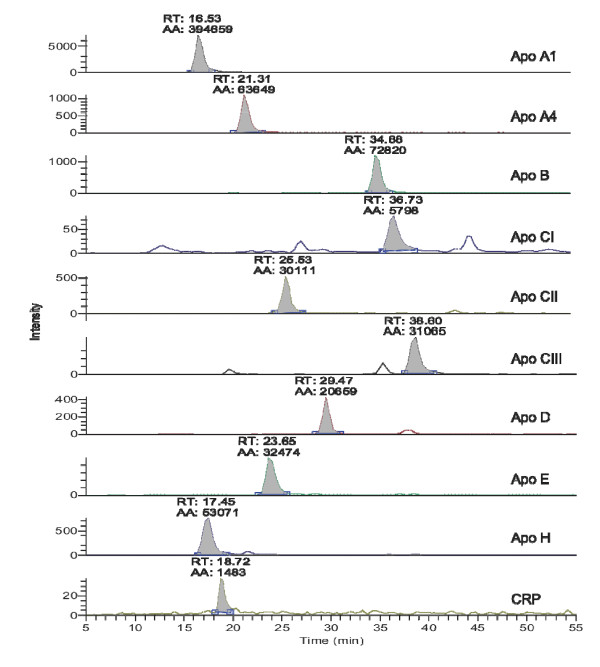
**Biomarker assay integration utilizing high performance nano-separation (RT: retention time, AA: peak area, using automatic integration)**.

The intention of developing the cardiovascular SRM-assay is to manage quantitative read-outs for these ten biomarkers with a 30-minute cycle time. The resulting high-resolution chromatographic nano-separation of the cardiovascular assay developed, is depicted in Figure 2. Isotope labeled target peptide are synthesized by C13 inclusion, and used as the internal standards for absolute quantitations, as indicated by the asterisk at a given amino acid position (see Table 1).

**Figure 3:**
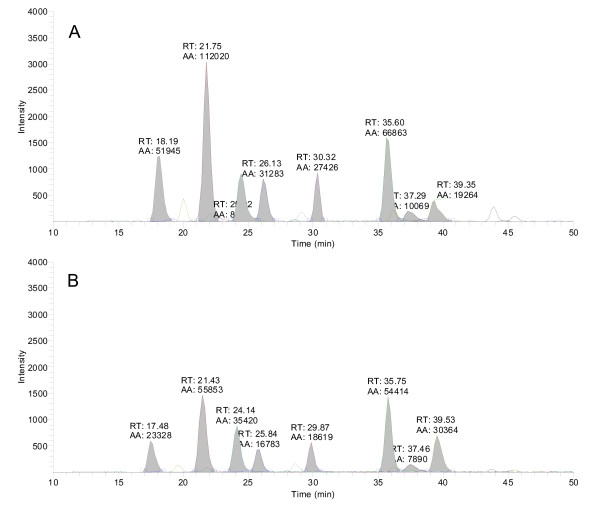
**Extracted ion chromatograms of the Apolipoprotein assay in an LC-MS/MS analysis of pooled male (A) and female (B) plasma tryptic digest (RT: retention time, AA: peak area, using automatic integration)**.

Applying the cardiovascular assay to biobank or other clinical study patient samples will require a validation step, where sample matrix variations are investigated. This is typically performed by choosing age- and sex-matched samples. In Figure 3A and 3B, corresponding spectra are presented from hospital subjects, and their respective cardiovascular biomarker levels in blood plasma. These two analysis runs (Figure 3A and 3B), are read-outs from two pooled samples with blood sampling made from 10 individuals. These examples were taken from a pooled cohort of age-grouped men (group 25-45 and 45-65, respectively) in Figure 3A.

## Biomarker Disease Mechanisms within Prostate Cancer

Prostate cancer is one of the fastest developing foci within disease areas with high unmet needs. Biomarker research within this field has been intense and productive within the last decade [31-34]. The prostate specific antigen (PSA) is a biomarker for disease indication that has been used world wide with both positive and negative outcomes. The reason for the shortcoming of this diagnostic measure and assay is not entirely clear. In our research team, we have been studying the alteration of PSA for many years in order to understand the relationship between PSA presence levels and disease progression [35]. One of our strategies has been to identify as many PSA-isoforms as possible, in order to link the quantitation with qualitative analysis. Proteomics data generated from more than a thousand prostate sequencing experiments [35,36], posed a major challenge to bioinformatics evaluations, utilizing databases we built in collaborative efforts (unpublished data), as well as annotations with Mascot, X!Tandem and Sequest (Végvári Á, Rezeli M, Sihlbom C, Häkkinen J, Carlsohn E, Malm J, Lilja H, Marko-Varga G, Laurell T: Mass Spectrometry Reveals Molecular Microheterogeneity of Prostate Specific Antigen in Seminal Fluid, submitted). By the nine PSA-forms we identified until today (Végvári Á, Rezeli M, Sihlbom C, Häkkinen J, Carlsohn E, Malm J, Lilja H, Marko-Varga G, Laurell T: Mass Spectrometry Reveals Molecular Microheterogeneity of Prostate Specific Antigen in Seminal Fluid, submitted), it is clear in our experience that the details of any given target, such as PSA in our case, the bioinformatics data at hand, and the "*in silico*" predictions that are experimentally verified, are powerful combinations. It allows us to reach statistical power with significance scoring in clinical situations that previously have been unknown.

As an outcome of these recent findings, we are aiming at profiling the PSA-isoforms present in clinical biofluids with new technologies such as SRM. These assays will be run in parallel to the standard measurements performed by ELISA used in clinical practice today Mass spectrometry with high-resolving nano-separation is a technique that we have developed specific methods and assays around [37,38].

**Figure 4:**
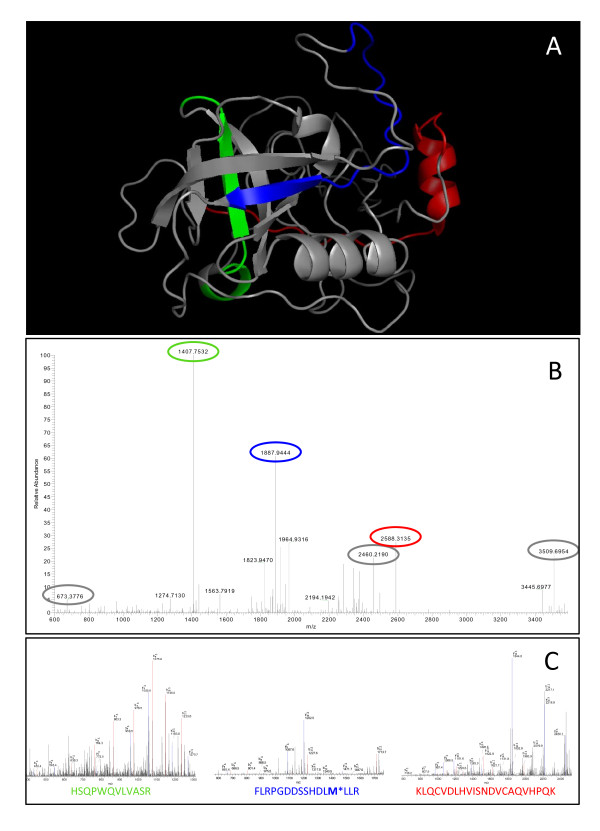
I**llustration of PSA identification in clinical samples by mass spectrometry-based proteomic analysis**. (A) The molecular structure of PSA with three typical tryptic peptides used for identification by sequences (colored regions). Mass spectra generated from molecular forms of PSA by both (B) high resolving FT full and (C) corresponding MS/MS fragmentation scans of those tryptic peptides highlighted with the same colors.

PSA is a small glycoprotein with five disulphide bridges (Mw = 28 kDa), constituting 4 helices and 6 beta strands densely as illustrated in Figure 4A. The colored parts of the crystal structure in Figure 4A are indicating the sequence areas of the target, which corresponds to the MS-sequences generated, in order to identify the nine PSA-forms, found in clinical samples (Végvári Á, Rezeli M, Sihlbom C, Häkkinen J, Carlsohn E, Malm J, Lilja H, Marko-Varga G, Laurell T: Mass Spectrometry Reveals Molecular Microheterogeneity of Prostate Specific Antigen in Seminal Fluid, submitted).

The resulting mass spectra generated from PSA molecular forms are presented in Figure 4B, where the different sequence masses are depicted. Figure 4B provides the full mass spectrum of PSA isolated during a separation step. The resulting spectrum identifies several tryptic peptides of PSA with high mass accuracy typical of FT analyzer of the Thermo LTQ Orbitrap. The MS-spectra presented in the figure caption (Figure 4B) typically had a <2 ppm accuracy, with a scoring factor of at least 30, but in many cases reaches statistical significance values of more than 100 (overall average score was 60). In addition, following a fragmentation process the sequences of these peptides are determined, and protein identification is attained with high confidence and accuracy. The corresponding MS/MS fragmentation spectra we generate in these screenings are shown in Figure 4B-D.

The entire set of PSA data were used in the development of our Prostate Database build, where we included a series of y- and c-ions, that were characteristic to each and every PSA form identified.

## Conclusions

The field of proteomics is currently undergoing a major development phase. Technology platforms have been developed to achieve high capacity assay capabilities by combining high-resolution nano-separations with mass spectrometry quantitation to deliver the basis for multiplex protein diagnosis.

Correlation of biomarker quantitations with patient demographics, clinical measurement data, such as imaging technologies as computed tomography (CT), and clinical outcome data are posed to provide a monitoring of disease progression as well as treatment response.

The development of standardized methods for measuring novel biomarkers associated with the most widespread diseases is being approached from a variety of methods including the screening of individual biomarkers in multiplex formats such as the SRM assay. The SRM platform also opens up for an option to provide patients with opportunities for improved personalized therapeutic alternatives [39,40]. As an example, Posttranslational modifications are well known resulting outcomes of protein rearrangements that occurs within disease mechanisms. Typically, phosphorylation alterations upon activations have been developed for instance within the signaling cascade of event of kinases, as well as glycosylation alterations for instance in cancer.

Nitro proteins have become the new PTM finding with a clear link to disease. It was observed, especially in lung cancers and brain tumors, among others that nitrification mechanisms were advancing as a cellular unregulated activity [41,42]. One of the current objectives is to map out and discover many novel endogenous nitro proteins, and link it to disease and disease progression. In this respect, biological action of reactive oxygen species (ROS), reactive nitrogen species (RNS), and oxidative stress are central biological effects that seem to have attracted specific interest [41,42]. It is also envisioned that the global initiatives on biobanking will play a major role in the near future where it is expected that clinical biomaterial derived from patients will earn be a good investment to serve as a deposit of medical interest in the form of knowledge and therapies that can be built and grow out of a Biobank archive.

## Abbreviations

COPD: Chronic obstructive pulmonary disease; FT: Fourier transformation; HUPO: Human Proteome Organization; HPP: Human Proteome Project; MRM: Multiple reaction monitoring; SRM: Single reaction monitoring; PTM: Posttranslational modification; ROS: Reactive oxygen species; RNS: Reactive nitrogen species

## Competing interests

The authors declare that they have no competing interests.

## Authors' contributions

The authors contributed equally to this work. All authors read and approved the final manuscript.

## References

[B1] Hood L Heath JR Phelps ME Lin BY Systems biology and new technologies enable predictive and preventative medicine Science 2004 306 640 643 10.1126/science.1104635 15499008

[B2] Marko-Varga G Lindberg H Lofdahl CG Jonsson P Hansson L Dahlback M Lindquist E Johansson L Foster M Fehniger TE Discovery of biomarker candidates within disease by protein profiling: Principles and concepts J Proteome Res 2005 4 1200 1212 10.1021/pr050122w 16083270

[B3] Prevention and Control of Chronic Respiratory Diseases at Country Level - Towards a Global Alliance against Chronic Respiratory Diseases (GARD) http://www.who.int/respiratory/publications/WHO_NMH_CHP_CPM_CRA_05.1.pdf

[B4] Taylor CF Paton NW Lilley KS Binz PA Julian RK Jones AR Zhu WM Apweiler R Aebersold R Deutsch EW The minimum information about a proteomics experiment (MIAPE) Nat Biotechnol 2007 25 887 893 10.1038/nbt1329 17687369

[B5] Taylor CE Minimum reporting requirements for proteomics: A MIAPE primer Proteomics 2006 39 44 10.1002/pmic.200600549 17031795

[B6] Wright JC Hubbard SJ Recent Developments in Proteome Informatics for Mass Spectrometry Analysis Comb Chem High T Scr 2009 12 194 202 10.2174/138620709787315508 19199887

[B7] Orchard S Jones A Albar JP Cho SY Kwon KH Lee C Hermjakob H Tackling Quantitation: A Report on the Annual Spring Workshop of the HUPO-PSI Proteomics 2010 10 3062 3066 10.1002/pmic.201090075 20806224

[B8] Baker MS Building the 'practical' human proteome project - The next big thing in basic and clinical proteomics Curr Opin Mol Ther 2009 11 600 602 20077630

[B9] Hochstrasser D Should the Human Proteome Project Be Gene- or Protein-centric? J Proteome Res 2008 7 5071 5071 10.1021/pr800884r 19367715

[B10] A Gene-centric Human Proteome Project Mol Cell Prot 2010 9 427 429 10.1074/mcp.H900001-MCP200 PMC2830851 20124355

[B11] Atkinson AJ Colburn WA DeGruttola VG DeMets DL Downing GJ Hoth DF Oates JA Peck CC Schooley RT Spilker BA Biomarkers and surrogate endpoints: Preferred definitions and conceptual framework* Clin Pharmacol Ther 2001 69 89 95 10.1067/mcp.2001.113989 11240971

[B12] Marko-Varga G Ogiwara A Nishimura T Kawamura T Fujii K Kawakami T Kyono Y Tu HK Anyoji H Kanazawa M Personalized medicine and proteomics: Lessons from non-small cell lung cancer J Proteome Res 2007 6 2925 2935 10.1021/pr070046s 17636986

[B13] Lander ES Linton LM Birren B Nusbaum C Zody MC Baldwin J Devon K Dewar K Doyle M FitzHugh W Initial sequencing and analysis of the human genome Nature 2001 409 860 921 10.1038/35057062 11237011

[B14] Venter JC Adams MD Myers EW Li PW Mural RJ Sutton GG Smith HO Yandell M Evans CA Holt RA The sequence of the human genome Science 2001 291 1304 1351 10.1126/science.1058040 11181995

[B15] Collins FS Morgan M Patrinos A The human genome project: Lessons from large-scale biology Science 2003 300 286 290 10.1126/science.1084564 12690187

[B16] Jasny BR Roberts L Unlocking the genome Science 2001 294 81 81 10.1126/science.294.5540.81 11588244

[B17] Human Proteome Project http://www.hupo.org/research/hpp/HPP_Jan_25_2010.pdf

[B18] Hancock W Omenn G LeGrain P Paik YK Proteomics, Human Proteome Project, and Chromosomes J Proteome Res 2011 10 210 210 10.1021/pr101099h 21114295

[B19] Aebersold R Auffray C Baney E Barillot E Brazma A Brett C Brunak S Butte A Califano A Celis J Report on EU-USA Workshop: How Systems Biology Can Advance Cancer Research (27 October 2008) Mol Oncol 2009 3 9 17 10.1016/j.molonc.2008.11.003 19383362 PMC2930781

[B20] Fehniger TE Marko-Varga G Clinical Proteomics Today J Proteome Res 2011 10 3 3 10.1021/pr1012569 21210716

[B21] Hood L A Personal Journey of Discovery: Developing Technology and Changing Biology Annu Rev Anal Chem 2008 1 1 43 10.1146/annurev.anchem.1.031207.113113 20636073

[B22] Anderson NL The Clinical Plasma Proteome: A Survey of Clinical Assays for Proteins in Plasma and Serum Clin Chem 2010 56 177 185 10.1373/clinchem.2009.126706 19884488

[B23] Anderson L Hunter CL Quantitative mass spectrometric multiple reaction monitoring assays for major plasma proteins Mol Cell Prot 2006 5 573 588 10.1074/mcp.M500331-MCP200 16332733

[B24] Hu Z Hood L Tan O Quantitative proteomic approaches for biomarker discovery Proteom Clin Appl 2007 1 1036 1041 10.1002/prca.200700109 21136755

[B25] Weston AD Hood L Systems biology, proteomics, and the future of health care: Toward predictive, preventative, and personalized medicine J Proteome Res 2004 3 179 196 10.1021/pr0499693 15113093

[B26] Lange V Picotti P Domon B Aebersold R Selected reaction monitoring for quantitative proteomics: a tutorial Mol Sys Biol 2008 4 Article number: 222 10.1038/msb.2008.61 PMC2583086 18854821

[B27] Hüttenhain R Malmström J Picotti P Aebersold R Perspectives of targeted mass spectrometry for protein biomarker verification Curr Opin Chem Biol 2009 13 518 525 19818677 10.1016/j.cbpa.2009.09.014 PMC2795387

[B28] Abbatiello SE Mani DR Keshishian H Carr SA Automated Detection of Inaccurate and Imprecise Transitions in Peptide Quantification by Multiple Reaction Monitoring Mass Spectrometry Clin Chem 2010 56 291 305 10.1373/clinchem.2009.138420 20022980 PMC2851178

[B29] Surinova S Schiess R Hüttenhain R Cerciello F Wollscheid B Aebersold R On the Development of Plasma Protein Biomarkers J Proteome Res 2011 10 5 16 10.1021/pr1008515 21142170

[B30] Ong SE Schenone M Margolin AA Li XY Do K Doud MK Mani DR Kuai L Wang X Wood JL Identifying the proteins to which small-molecule probes and drugs bind in cells Proc Natl Acad Sci USA 2009 106 4617 4622 10.1073/pnas.0900191106 19255428 PMC2649954

[B31] Finnskog D Järås K Ressine A Malm J Marko-Varga G Lilja H Laurell T High-speed biomarker identification utilizing porous silicon nanovial arrays and MALDI-TOF mass spectrometry Electrophoresis 2006 27 1093 1103 10.1002/elps.200500751 16523454

[B32] Klein RJ Hallden C Cronin AM Ploner A Wiklund F Bjartell AS Stattin P Xu JF Scardino PT Offit K Blood Biomarker Levels to Aid Discovery of Cancer-Related Single-Nucleotide Polymorphisms: Kallikreins and Prostate Cancer Cancer Prev Res 2010 3 611 619 10.1158/1940-6207.CAPR-09-0206 PMC2865570 20424135

[B33] Steuber T Vickers AJ Serio AM Vaisanen V Haese A Pettersson K Eastham JA Scardino PT Huland H Lilja H Comparison of free and total forms of serum human kallikrein 2 and prostate-specific antigen for prediction of locally advanced and recurrent prostate cancer Clin Chem 2007 53 233 240 10.1373/clinchem.2006.074963 17185368

[B34] Vickers AJ Cronin AM Roobol MJ Savage CJ Peltola M Pettersson K Scardino PT Schroder FH Lilja H A Four-Kallikrein Panel Predicts Prostate Cancer in Men with Recent Screening: Data from the European Randomized Study of Screening for Prostate Cancer, Rotterdam Clin Chem Res 2010 16 3232 3239 10.1158/1078-0432.CCR-10-0122 PMC2891337 20400522

[B35] Végvári Á Rezeli M Welinder C Malm J Lilja H Marko-Varga G Laurell T Identification of Prostate Specific Antigen (PSA) Isoforms in Complex Biological Samples Utilizing Complementary Platforms J Proteomics 2010 73 1137 1147 20102753 10.1016/j.jprot.2010.01.008 PMC2856840

[B36] Végvári Á Rezeli M Sihlbom C Carlsohn E Malm J Lilja H Laurell T Marko-Varga G Marko-Varga G, Simones T Characterization of PSA in Clinical Samples by Mass Spectrometry4th EuPA Scientific Meeting - A Proteomics Odyssey Towards Next Decades; Estoril, Portugal 2010 Ook-Press Ltd 508 510

[B37] Végvári Á Marko-Varga G Clinical Protein Science and Bioanalytical Mass Spectrometry with an Emphasis on Lung Cancer Chem Rev 2010 110 3278 3298 20415473 10.1021/cr100011x

[B38] Choudhary C Mann M Decoding signalling networks by mass spectrometry-based proteomics Nat Rev Mol Cell Biol 2010 11 427 439 10.1038/nrm2900 20461098

[B39] Kiyonami R Domon B Selected reaction monitoring applied to quantitative proteomics Methods Mol Biol 2010 658 155 166 full_text 20839103 10.1007/978-1-60761-780-8_9

[B40] Picotti P Rinner O Stallmach R Dautel F Farrah T Domon B Wenschuh H Aebersold R High-throughput generation of selected reaction-monitoring assays for proteins and proteomes Nat Methods 2010 7 43 U45 10.1038/nmeth.1408 19966807

[B41] Zhan XQ Desiderio DM The human pituitary nitroproteome: detection of nitrotyrosyl-proteins with two-dimensional Western blotting, and amino acid sequence determination with mass spectrometry Biochem Biophys Res Commun 2004 325 1180 1186 10.1016/j.bbrc.2004.10.169 15555551

[B42] Zhan XQ Desiderio DM The use of variations in proteomes to predict, prevent, and personalize treatment for clinically nonfunctional pituitary adenomas The EPMA Journal 2010 1 439 459 10.1007/s13167-010-0028-z PMC3405333 23199087

